# Arsenic and the Pharmacopœia

**Published:** 1904-09-17

**Authors:** 


### The Hospital.

A JOURNAL OF

The Medical Sciences and Hospital Administration.

VOL. XXXVI.—No. 938. SEPTEMBER 17, 1904.

### ARSENIC AND THE PHARMACOPŒIA.

WHEN, owing to the insight and ability of Dr. Ernest S. Reynolds, it was proved, some four years ago, that a widespread outbreak of peripheral neuritis which occurred in the North of England was due to the presence of arsenic in beer, the far-reaching effects of the discovery could hardly have been anticipated. There were various immediate results by no means unimportant to the individuals concerned, but the first public indication of the significance of Dr. Reynolds's observation was the [illegible]pointment of a Royal Commission to investigate the whole subject of arsenical poisoning. The evidence produced before this Commission showed, much no doubt to the surprise of the public, that arsenic in small quantities is present in an enormous number of substances which differ widely from one another both in their sources and in the purposes for which they are employed. For example, malt, hops, chicory, dried orange and lemon peel, tobacco, quinine sulphate, and wool fat have all, by competent authorities, been placed under suspicion. One outcome from all this was the startling suggestion that small quantities of arsenic introduced into the body over long periods in various foods or systematically administered for medicinal purposes might reasonably be held responsible for the increase of cancer in the community. This suggestion, though advanced with a considerable degree of courage, has not made much progress in the opinion of the medical profession, and in recent months little has been heard of it. A more direct and legitimate consequence of the work of the Royal Commission was the proposal that the tests for the detection of arsenic should be scrutinised in regard to their efficiency as methods for the detection of the poison in small quantities. This manifestly is a point which has a special bearing on the presence of arsenic in medicinal substances, for the purity of these is determined by the official standards of the Pharmacopœia, and the evidence submitted to the Royal Commission raised the question whether these standards did in fact secure in a number of instances a sufficiently severe test. To determine this question a series of investigations has been conducted by Professor Wyndham Dunstan at the instigation of the Pharmacopœia Committee of the General Medical Council. The object has been to secure tests which could readily be applied by a properly qualified pharmaceutical chemist, and which, whilst fixing a standard of practical purity, should not place this at a level only capable of attainment at great cost and expense. Professor Dunstan's report, which has recently been issued, states that none of the existing official tests are specified with the precision requisite to fix a definite standard. On the other hand, it is recognised that the Marsh-Berzelius test, though the most delicate of all known methods, requires such complicated apparatus and such extreme purity in the reagents employed as to render it unsuitable for the demands of the Pharmacopœia Committee. What is advised therefore is the adoption of the test proposed by Mayençon and Bergeret, which depends on the production by arseniuretted hydrogen of a stain on paper soaked in mercuric chloride. By means of a set of standard stains representing respectively one, two, three, and four parts per million, the significance of a stain obtained from a known quantity of any drug can be determined. It is suggested that for the majority of drugs which are administered in small quantities the maximum allowance of arsenical contamination should not exceed the equivalent of three parts of arsenium per million, which corresponds roughly to $\frac{1}{36}$ grain of white arsenic in a pound. In the case of citric and tartaric acids, which are largely used in foods and drinks, a higher standard is suggested—namely, less than one part of arsenium in a million parts of the drug. A somewhat stricter demand is also made in regard to sulphuric, hydrochloric, and nitric acids, and to solution of ammonia, as these are largely used in the manufacture of various medicinal preparations. This brief statement includes the principles on which Professor Dunstan insists, but their application to the various drugs has demanded a large amount of chemical work the details of which are clearly set forth in the report he has framed. In addition, he advises that the list of official substances defined as giving "no characteristic reaction with the tests for arsenium" should be extended by the inclusion in it of no less than 41 additional pharmacopœial remedies. This in itself is sufficient to illustrate how widely diffused is the presence of arsenic, and how difficult or even impossible it is to secure absolute freedom from it. Apart from its specific value Professor Dunstan's report has this interest, that it illustrates how sustained are the oversight and labour which issue in the production of a national pharmacopœia and in the maintenance and elevation of its standards. Between successive editions long years elapse which to the unthinking may seem years of rest and hibernation. But the very opposite is the case. No sooner is a new edition born than critics and circumstances show the need for improvement. It is in supervising and guiding the methods and opportunities of such improvement that the largely unrecognised labours of the Pharmacopœia Committee are occupied. And it is no small part of the justification of the existence of the General Medical Council that, though silently, not the less effectually, it conserves the public welfare by promoting and directing work on which an extension of the efficiency and purity of substances employed as medicines essentially depends.

